# 1，3-二油酸-2-棕榈酸甘油三酯纯度标准物质定值技术

**DOI:** 10.3724/SP.J.1123.2025.10031

**Published:** 2026-07-08

**Authors:** Xinyu WANG, Ying WANG, Mengqian XU, Xia ZHOU, Hongtao CHU, Qinghe ZHANG, Xiuqin LI

**Affiliations:** 1. 齐齐哈尔大学化学与化学工程学院，黑龙江 齐齐哈尔 161006; 1. College of Chemistry and Chemical Engineering，Qiqihar University，Qiqihar 161006，China; 2. 中国计量科学研究院化学计量与分析科学研究所，北京 100029; 2. Division of Chemical Metrology and Analytical Science，National Institute of Metrology，Beijing 100029，China; 3. 国家市场监督管理总局重点实验室（营养与健康化学计量及应用），北京 100029; 3. Key Laboratory of Chemical Metrology and Applications on Nutrition and Health，State Administration for Market Regulation，Beijing 100029，China

**Keywords:** 1，3-二油酸-2-棕榈酸甘油三酯, 纯度标准物质, 定值技术, 高效液相色谱-电雾式检测器, 定量核磁共振波谱法, 1，3-oleic-2-palmitic triglyceride （OPO）, purity reference materials, value assignment, high performance liquid chromatography-charged aerosol detector （HPLC-CAD）, quantitative nuclear magnetic resonance （qNMR）

## Abstract

1，3-二油酸-2-棕榈酸甘油三酯（OPO）作为婴幼儿配方乳粉中允许添加的营养强化剂受到了广泛关注，亟需研制高纯标准物质以满足相关检测的计量溯源要求。本文系统研究了用于OPO标准物质研制的质量平衡法与定量核磁共振波谱法两种不同原理的定值技术。质量平衡法中，采用高效液相色谱-电雾式检测器对4个结构类似的微量杂质准确定量，同时对原料中的水分、挥发性有机溶剂残留、不挥发性杂质等进行定量分析，通过杂质扣减法得到OPO纯度为98.51%；定量核磁共振波谱法中，对氘代溶剂、内标物、定量峰进行优化，选择化学位移2.33（OPO）和6.88（尼泊金乙酯）作为定量峰对主成分含量直接测定，得到OPO纯度为98.82%；两种定值方法准确可靠，定值结果一致性好，可用于对OPO纯度标准物质的定值。同时对OPO标准物质候选物的均匀性、稳定性进行监测，对定值方法以及不均匀性和不稳定性引入的不确定度进行了评估。结果表明：OPO纯度标准值为98.7%，相对扩展不确定度为0.7%（*k*=2，*k*为包含因子），该标准物质的成功研制为相关检测提供了溯源标准。

## Abstract (en)

1，3-Oleic-2-palmitic triglyceride （OPO） has received widespread attention as a nutritional fortifier allowed to be added to infant formulas， and there is an urgent need to develop high-purity certified reference material （CRM） to meet the metrological traceability requirements of relevant tests. Value assignment methods for pure materials are a key technical challenge in the development of high-purity CRMs. The commonly used mass balance （MB） method indirectly determines the purity of the material by subtracting impurities， which involves the accurate quantification of structurally similar impurities， moisture， volatile solvents， and non-volatile impurities. Among these steps， the separation and analysis of structurally similar impurities of the main component are the key to MB method. Owing to the lack of ultraviolet/fluorescence chromophores， OPO cannot be effectively detected by liquid chromatography with terminal absorption （190–210 nm）； this technique lacks sufficient sensitivity for low-level related impurities. Therefore， with a universal detector， this study established a high performance liquid chromatography-charged aerosol detector （HPLC-CAD） method for the analysis of structural analogue impurities in OPO， and applied the mass balance method to certify the purity of the reference material. On the other hand， based on quantitative nuclear magnetic resonance （qNMR） technology， a qNMR purity assignment method for OPO purity was established by optimizing key parameters such as NMR internal standards and quantitative peaks. In the mass balance method， an HPLC-CAD was selected as the core technique for quantifying trace structurally similar impurities of OPO. The chromatographic conditions were as follows： Nova Pak C8 column （150 mm×3.9 mm， 4 μm）， acetonitrile-isopropanol （90∶10， volume ratio） at a flow rate of 1.0 mL/min， sample dissolved in *n*-hexane-isopropanol （1∶1， volume ratio） at a mass concentration of 2.0 mg/mL with an injection volume of 5 μL. Under these conditions， four structurally related trace impurities were baseline-separated and accurately quantified. Method validation showed that in the mass concentration range of 0.001-0.025 mg/mL， good linear relationship was obtained （*R*²=0.999 1）. The limits of detection （LOD） and quantification （LOQ） were 0.000 5 mg/mL and 0.001 mg/mL， respectively， meeting the requirements for quantitative analysis. Six replicate measurements gave an average total impurity content of 1.23% （standard deviation 0.03%）， corresponding to an OPO main component content of 98.77%. In addition， Karl Fischer coulometric titration was used to determine moisture content， headspace gas chromatography-flame ionization detection （HS-GC-FID） was used to analyze residual volatile organic solvents （VOC）， and thermogravimetric analysis was used to determine non-volatile impurities. The purity of OPO was calculated as 98.51% by subtracting related impurities， moisture， VOC and non-volatile residues. In qNMR， direct and accurate quantification of the main component was achieved by optimizing key parameters： deuterated chloroform was selected as the solvent； ethyl paraben was used as the internal standard； selected chemical shifts of 2.33 （OPO） and 6.88 （ethyl paraben） as quantitative peaks. Accurately weigh approximately 10.0 mg of OPO and 3.0 mg of the internal standard in a brown bottle， dissolved in 0.50 mL of deuterated chloroform， and transferred to a 5 mm NMR tube. After six parallel measurements， the average purity of OPO was calculated to be 98.82%， with a standard deviation of 0.21%. Additionally， the homogeneity and stability of the candidate high-purity OPO reference material were evaluated. The uncertainties introduced by value assignment， inhomogeneity， and instability were evaluated. The results showed that the certified purity value of OPO was 98.7%， with a relative expanded uncertainty of 0.7% （*k*=2，where *k* is the coverage factor corresponding to a confidence probability of approximately 95% under the normal distribution）. The successful development of this certified reference material provides a traceability standard for relevant detections and fills the gap in OPO purity reference material.

母乳中60%～70%的棕榈酰基位于甘油三酯的*sn*-2位，而*sn*-1和*sn*-3位基本为油酰基和亚油酰基，1，3-二油酸-2-棕榈酸甘油三酯（OPO）和1-油酸-2-棕榈酸-3-亚油酸甘油三酯（OPL）是母乳中主要的USU（U不饱和脂肪酸，S饱和脂肪酸）型甘油三酯。OPO进入小肠后，*sn*-1和*sn*-3位油酰基优先被水解生成游离的油酸，而*sn*-2位棕榈酰基不易被水解仍然与甘油骨架连接形成棕榈酸单甘油酯，二者均很容易被人体吸收，OPO这一吸收优势依赖其结构特征；若棕榈酰基主要连接在*sn*-1和*sn*-3位，水解后生成的游离棕榈酸容易与钙结合降低水溶性而被排出体外，造成能量吸收不足、钙等矿物质流失以及引起便秘等^［1-6］^。因此，OPO常作为营养强化剂添加在婴幼儿配方乳粉中，以弥补婴幼儿配方奶粉与母乳之间甘油三酯成分的差异。婴幼儿配方乳粉等样品中OPO的分析检测成为相关产品质量控制与市场监管的必然需求。标准物质是分析检测方法开发以及结果准确性保证的基础，虽然国内外关于OPO合成制备的研究报道众多，化学纯原料也实现了商品化，但是高纯标准物质国内外尚属空白，无法保证分析检测结果的计量溯源性。

纯度定值分析是高纯标准物质研制的关键技术难点，常用的质量平衡定值方法（mass balance，MB）是通过准确测定结构类似物杂质、水分、挥发性溶剂以及不挥发性杂质的含量，采用杂质扣减的方法^［7］^，间接获得标准物质的纯度，其中主成分结构类似物杂质的分离分析是该定值分析方法的关键。OPO由于没有紫外及荧光等发光基团，采用末端吸收（190~210 nm）的液相色谱检测技术灵敏度不够，无法检测到含量较低的相关杂质^［8］^，因此本研究基于通用型检测器，建立了OPO主成分及结构类似杂质的高效液相色谱-电雾式检测器（HPLC-CAD）分析方法，采用MB法对标准物质纯度进行定值分析。另一方面，基于定量核磁共振波谱法（quantitative nuclear magnetic resonance， qNMR），通过对内标、定量峰等关键参数的优化^［9，10］^，建立了OPO纯度的qNMR定值分析方法。

## 1 实验部分

### 1.1 主要仪器与试剂

SCIEX Zeno TOF 7600型质谱仪（美国AB SCIEX公司）；AVANCE Ⅲ型400 MHz核磁共振仪（瑞士Bruker公司）； Thermo Ultimate 3000 DGLC型液相色谱仪串联电雾式检测器（美国Thermo公司）；Perkin Elmer Pyris 1 TGA热重分析仪（美国Perkin Elmer公司）；C30S型卡尔费休滴定仪和UMX2型电子天平（瑞士Mettler Toledo公司）；6890N G1888型气相色谱-氢火焰离子化检测器-顶空进样器（美国Agilent公司）。

乙腈：色谱纯（Merck试剂）；实验用水：重蒸馏水；甲酸铵：色谱纯（上海麦克林生化科技股份有限公司）；异丙醇、正己烷：色谱纯（美国Fisher公司）；乙酸钠：99.8%（上海阿拉丁生化科技股份有限公司）；氘代氯仿：99.8%（北京百灵威科技有限公司）；尼泊金乙酯（GBW06120）：99.97%±0.05%（中国计量科学研究院）；OPO标准物质候选物：由第三方公司制备，标示纯度>99%。

### 1.2 实验方法

#### 1.2.1 OPO标准物质候选物定性分析方法

核磁共振分析：激发脉冲角度zg90°，采样时间4.0 s，时间域数据点64K，扫描谱宽（SWH）20.00，激发中心（O1P）4.61，扫描宽度8 000.256 Hz，弛豫延迟（D1）23.52 s，累计采样128次，探头温度299.0 K，偏置频率2 985.12 Hz，接收增益128。

电子活化解离高分辨质谱分析（EAD-HRMS）：采用电喷雾离子源，正离子扫描，扫描模式为信息依赖采集（information-dependent acquisition，IDA）模式；电喷雾电压：+5 500 V；离子源温度：300 ℃；EAD的电子动能（KE）设置为10 eV；电子束电流为7 000 nA；扫描范围为*m/z* 100~900。进样方式：针泵直接进样。

#### 1.2.2 质量平衡法纯度定值

结构类似物杂质分析 采用HPLC-CAD进行分析，选择Nova-Pak C8色谱柱（150 mm×3.9 mm，4 µm）。流动相：乙腈-异丙醇（90∶10，体积比）。样品质量浓度：2.0 mg/mL，溶剂：正己烷-异丙醇溶液（1∶1，体积比）；进样体积：5 μL。

水分分析 采用卡尔费休库仑滴定法进行分析，样品混合时间为15 s；最大起始漂移值25 μg/min，终点100.0 mV，采用直接进样方式。采用水分标准物质校准仪器后，称取10 mg OPO样品进样测试，待滴定结束后记录水分含量。

不挥发性杂质分析 采用热重法进行分析，OPO取样量约10 mg，升温程序如下：初始30 ℃，匀速升温至80 ℃，保持10 min，再由80.00 ℃升温至110.00 ℃后保持10 min，最后由110.00 ℃升温至850.00 ℃并保持10 min。升温速度始终为10.00 ℃/min。根据灼烧前后重量的变化，计算不挥发性杂质含量。

挥发性有机溶剂残留分析 采用顶空气相色谱法分析，Agilent DB-624色谱柱（30 m×0.320 mm×1.80 μm）；顶空参数：加热箱（恒温炉）温度90 ℃，定量环温度115 ℃，传输线温度120 ℃，样品平衡时间15 min；进样量1 mL，不分流进样；载气（氮气）1.0 mL/min，氢气40 mL/min，空气400 mL/min；进样口温度130 ℃；检测器温度260 ℃；升温程序：初始40 ℃保持5 min，以4 ℃/min升温至100 ℃，保持1 min，再以30 ℃/min升温至220 ℃，保持1 min，运行时间26 min。

#### 1.2.3 qNMR法纯度定值

选择尼泊金乙酯为内标物，分别准确称量约10.0 mg OPO样品和3.0 mg内标物置于同一棕色玻璃瓶，在0.50 mL氘代氯仿中振荡完全溶解后，转移至直径为5 mm的核磁管中待测。

qNMR测定参数同1.2.1节NMR分析参数，OPO与尼泊金乙酯的定量峰化学位移分别为2.33和6.88。

## 2 结果与讨论

### 2.1 OPO主成分定性分析

#### 2.1.1 EAD-HRMS质谱定性

图1为OPO标准物质候选物在EAD裂解模式下的二级质谱图，由质谱图可得出［M+Na］^+^的分子质量为881.74 Da，与其理论相对分子质量（859.415）相符合。在EAD碎裂模式中，产生特征碎片离子*m/z* 305.24，由此可以判断出在甘油母核*sn*-2位连接的是C16：0的脂肪酸酰基链，而在*sn*-1/*sn*-3位特征碎片的位置也能看到*m/z*为345.28和347.25的离子，表明在*sn*-1位和*sn*-3位连接的都是C18：1脂肪酸酰基链；母离子［M+Na］^+^丢失*sn*-1、*sn*-2、*sn*-3任意位置的1个脂肪酸酰基链C18：1（O）脂肪酰基或者C16：0（P）脂肪酰基，均可产生［OO］^+^碎片*m/z* 625.52和［PO］^+^碎片*m/z* 599.50。在EAD碎裂模式中，脂肪酸酰基链可以产生连续的脱-CH_2_的碎片，而双键连接的位置则不会发生断裂，如图1所示，在C18：1链上自甲基端开始连续脱去7个-CH_2_（质量差14 Da）后，发现了质量差为26 Da的特征碎片，指示在C9、C10位间存在碳碳双键，且发现双键γ氢的存在，导致明显的麦氏重排发生，因此C11-C12单键断裂产生的碎片离子丰度最高，因此可以判断出C18：1脂肪酸酰基链所含有的C=C双键在C9位^［11］^。综上，候选物的质谱图与OPO的质谱裂解特征一致。

**图1:**
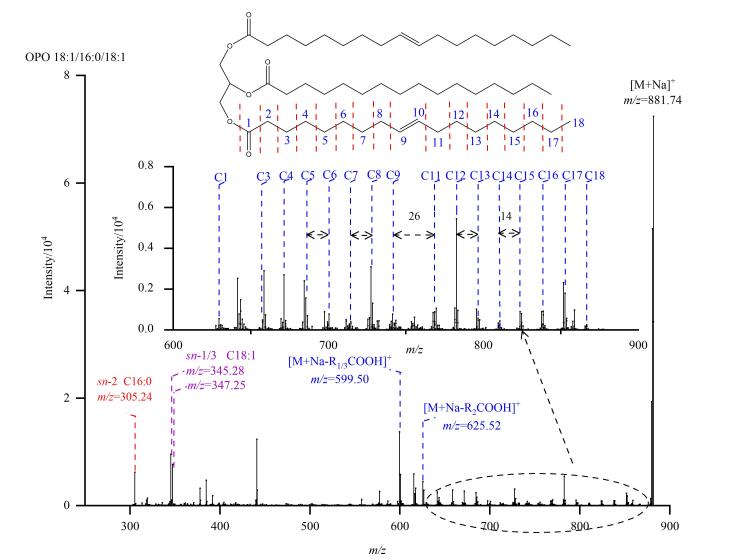
OPO标准物质候选物在EAD裂解模式下的二级质谱图

#### 2.1.2 核磁共振氢谱定性

图2为OPO标准物质候选物在氘代氯仿中的核磁共振氢谱图。由图可知，化学位移为7.26单峰为氘代氯仿的溶剂峰，甘油骨架B位置的氢化学位移为5.26（m，1H），甘油骨架上C和D位置上的氢分别产生4.29（dd，*J*=12.0，4.4 Hz，2H）和4.14（dd，*J*=12.0，6.0 Hz，2H）的化学位移。油酸酰基上双键的氢A的化学位移为5.34（m，4H），油酸酰基和棕榈酸酰基上羰基*α*位置上氢的化学位移为2.33（t，*J*=7.7 Hz，6H），烯丙位上氢的化学位移为2.03（q，*J*=5.8 Hz，8H），油酸酰基和棕榈酸酰基上羰基*β*位置G上氢的化学位移为1.63（m，6H），油酸酰基和棕榈酸酰基的CH_3_上氢I的化学位移为0.90（t，*J*=7.1 Hz，9H），剩余酰基链位置CH_2_的氢H产生1.28（m）的峰簇。

**图2:**
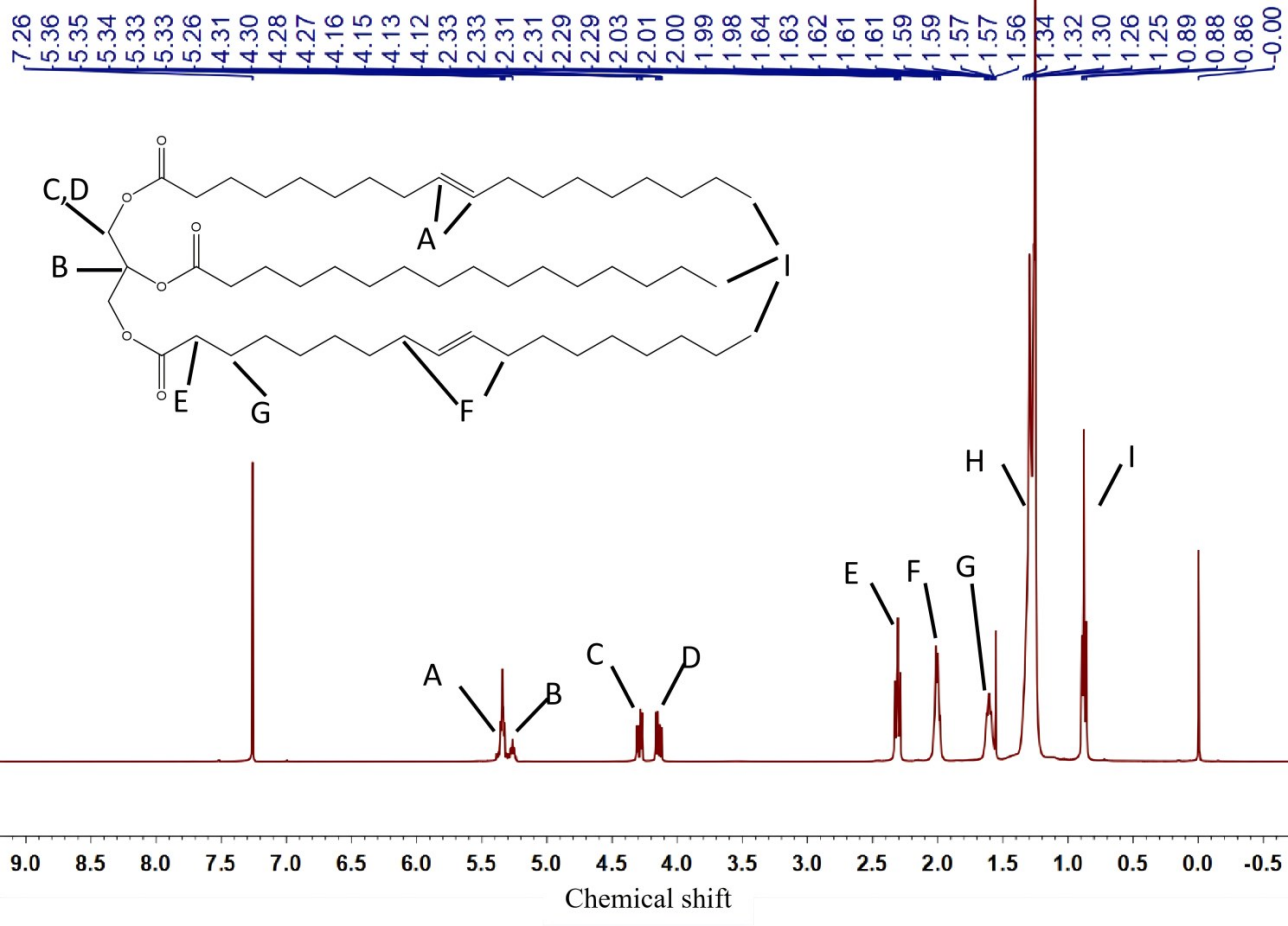
OPO标准物质候选物在氘代氯仿中的核磁共振谱图

实验测得的OPO样品的核磁共振氢谱图中氢原子的化学位移值与Scifinder软件模拟合成的OPO核磁共振氢谱图中氢原子的化学位移值基本一致，结合质谱定性结果，确认候选物为OPO。

### 2.2 质量平衡法纯度定值

#### 2.2.1 结构类似物杂质分析条件优化

OPO标准物质候选物在纯化制备过程中会产生一系列化学性质相近的、不易分离的结构类似物。然而OPO由甘油骨架、2个长链不饱和脂肪酸（油酰基）和1个棕榈酰基构成，缺乏发色基团无法产生紫外-可见吸收，因此只能选择通用型检测器进行分析，如CAD、蒸发光散射检测器（ELSD）等。同时甘油三酯极性非常小（lg *P*为10～15），在反相色谱的C18色谱柱上保留过强^［12，13］^，采用C8色谱柱较为常见，银离子正相色谱法（Ag^+^-HPLC）也常用来分析低极性的甘油三酯^［14-16］^，由于Ag^+^离子对双键的特异性吸附，Ag^+^-HPLC法能够很好地分析甘油三酯同分异构体。本研究考察了液相色谱检测器、色谱柱、流动相洗脱等条件，结果如下。

检测器与色谱柱的选择 分别采用CAD和ELSD两种检测器，比较了Chromspher 5 Lipids银离子色谱柱（250 mm×4.6 mm，5 µm）、Acquity UPLC BEH C18色谱柱（100 mm×2.1 mm，1.7 µm）和Nova-Pak C8色谱柱（150 mm×3.9 mm， 4 µm）对OPO的保留及对其结构相关杂质的分离能力，3种方法分别记为HPLC-ELSD-银离子柱、HPLC-ELSD-BEH C18柱、HPLC-CAD-Nova-Pak C8柱。结果表明：采用ELSD分析时，银离子正相色谱与C18反相色谱上仅能发现1个杂质，表明ELSD检测器的灵敏度较差，无法满足微量杂质的分析要求，同时OPO在C18柱上的保留时间过长。由图3可知，在CAD分析条件下可以分离出3个杂质，且基线平稳、峰形较好，因此本研究选取反相色谱串联CAD的方法，采用Nova-Pak C8色谱柱进行后续优化分析。

**图3:**
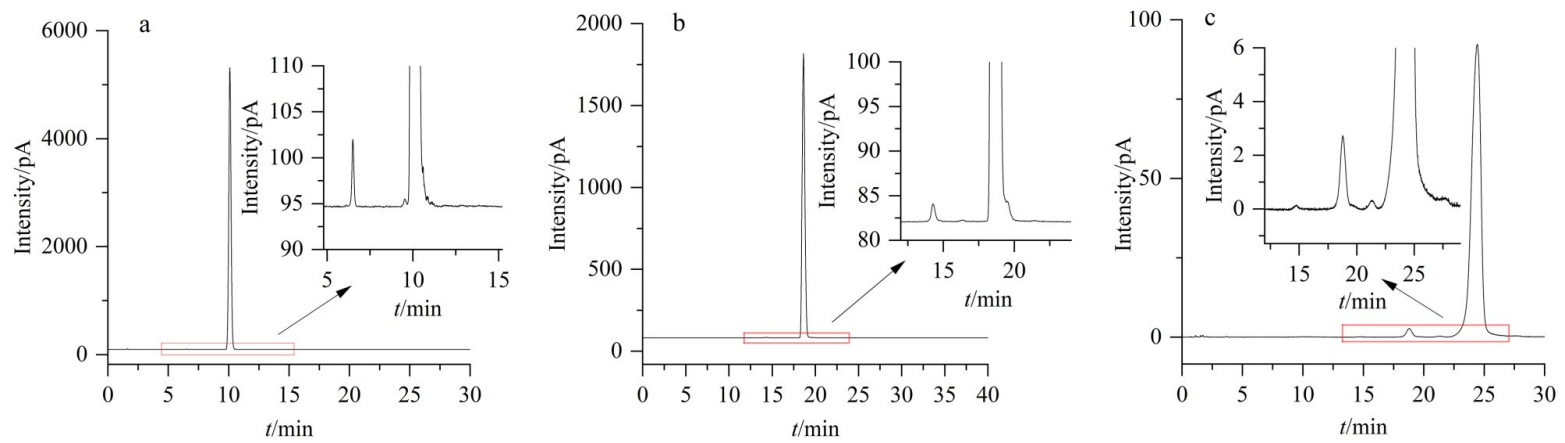
OPO在不同检测器和色谱柱等度洗脱下的液相色谱图

流动相优化 本研究选取异丙醇、乙腈、水3种溶剂，考察了条件Ⅰ（水-乙腈-异丙醇，10∶45∶45，体积比）和条件Ⅱ（乙腈-异丙醇， 90∶10，体积比）两种流动相体系在等度洗脱条件下对2.0 mg/mL OPO溶液的分离效果。由图4可知，流动相条件Ⅰ下发现3个明显杂质，主峰后面有痕量杂质，但由于该条件下洗脱速度较慢，该杂质扩散严重，峰宽过大，灵敏度降低，难以定量。在流动相条件Ⅱ下发现了4个杂质，主成分保留时间缩短至14.6 min，且该条件下基线平稳，所以选择乙腈-异丙醇作为流动相进行梯度优化。在条件Ⅱ的基础上，为了进一步缩短整体的分析时间，采用梯度洗脱程序进行分析，A相为乙腈，B相为异丙醇，起始流动相10%B，10 min内升至30%B，保留3 min，13~14 min降至10%B，平衡4 min（记为流动相条件Ⅲ），主成分保留时间由14.3 min缩短至9.8 min，结果表明，3号杂质无法与主成分得到较好分离。综上，选择条件Ⅱ作为定值条件。

**图4:**
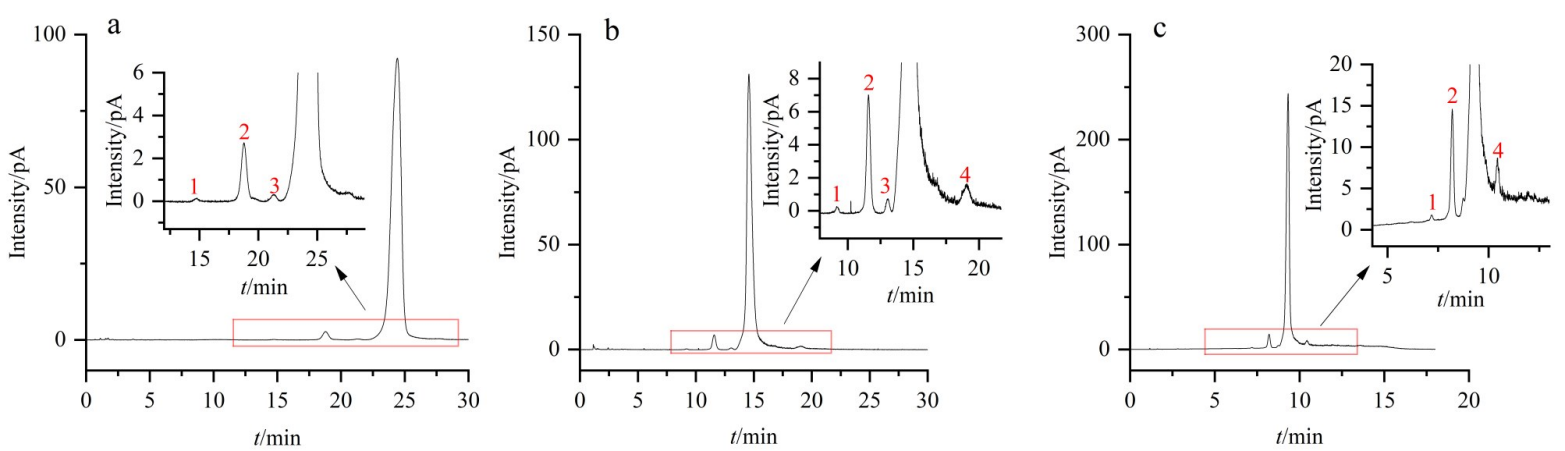
OPO在不同流动相条件下的液相色谱图

#### 2.2.2 方法的线性关系、检出限及定量限

配制一系列质量浓度的9种OPO标准溶液（0.001、0.005、0.01、0.02、0.05、0.1、0.2、0.5、1.0 mg/mL），采用HPLC-CAD法进行分析，以质量浓度-响应面积绘制标准曲线，由图5a可知在0.001～1.0 mg/mL范围内，方程为*y*=0.642 3*x* ^0.786 7^，该范围内不呈线性。由于CAD属于非依赖型检测器^［17］^，线性范围比较窄，在较大质量浓度范围内，响应曲线呈幂律关系而非线性，因此本研究选取0.001、0.002 5、0.006 25、0.012 5、0.025 mg/mL较窄范围的样品，绘制标准曲线。结果如图5b，在0.001～0.025 mg/mL范围内，线性方程为*y*=0.090 6*x*+0.084 2，相关系数（*R* ^2^）=0.999 1。进一步研究了0.001～1.0 mg/mL较大范围内，将质量浓度和峰面积均取对数绘制双对数标准曲线，由图5c可知，取双对数后，OPO在0.001 0～1.0 mg/mL范围内呈线性，*R* ^2^=0.999 1，表明采用HPLC-CAD时，也可采用双对数标准曲线进行定量校准，因此在窄范围校准曲线不适用时，可选择本校准方式进行定量。

**图5:**
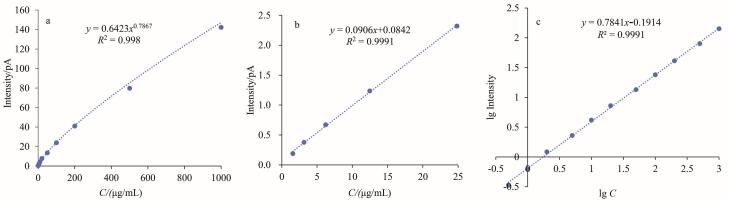
OPO的标准曲线图

方法的检出限以3倍信噪比确定，定量限以10倍信噪比确定。结果表明OPO在本分析方法下，检出限为0.000 5 mg/mL，定量限为0.001 mg/mL，表明分析方法灵敏度较好，能够满足结构类似物杂质的分析要求。

#### 2.2.3 结构类似物杂质分析

受CAD自身响应特性限制，对于高纯物质中微痕量杂质的分析无法使用传统的面积归一化方法进行主成分的直接定值。基于CAD对同类结构类似物可表现出相近响应的特点，采用一种物质对多个结构类似物进行定量分析。因此，本研究中采用OPO作为校准溶液，采用窄范围标准曲线法对候选物中的微量结构类似物杂质的含量进行测定^［18，19］^。结果见表1，由表可知总杂质的百分含量为1.23%，因此主成分的含量为98.77%。

**表1:** OPO样品中结构相关杂质含量测定结果

Sample No.	Sample content/（mg/mL）	Mass concentrations of impurities/（mg/mL）					Total impurity content/%	OPO content/%
		1	2	3	4	Total		
1	1.9686	0.00034	0.0191	0.00035	0.0046	0.0244	1.24	98.76
2	2.0062	0.00034	0.0190	0.00039	0.0049	0.0246	1.23	98.77
3	2.0721	0.00038	0.0205	0.00046	0.0038	0.0251	1.21	98.79
4	1.9892	0.00040	0.0193	0.00043	0.0038	0.0239	1.20	98.80
5	2.0204	0.00033	0.0198	0.00043	0.0040	0.0246	1.22	98.78
6	1.9878	0.00043	0.0204	0.00045	0.0043	0.0256	1.29	98.71
Average Standard deviation （SD）						0.0247	1.23	98.77
						0.0006	0.0003	0.0003

#### 2.2.4 质量平衡法定值结果

对随机抽取的6个OPO标准物质候选物，分别采用HPLC-CAD标准曲线法对结构相关杂质进行定量，主成分含量为98.77%，采用卡尔费休库仑滴定法测定水分含量为0.12%，热重法测定非挥发性杂质含量为0.14%，未测到挥发性溶剂，参照国家计量技术规范JJF1855-2020^［20］^的6.1节a法计算OPO的纯度，结果为98.51%。

### 2.3 qNMR纯度定值

#### 2.3.1 qNMR方法条件优化

本研究主要优化了激发脉冲角度、采集时间、时间域数据点、扫描宽度、弛豫延迟时间、增益数值、中心激发频率和累计采样次数，确定的参数最优条件见1.2.3节。在上述条件下，进一步对氘代溶剂、内标物、定量峰进行优化：内标物与氘代溶剂的选择要求内标物与待测分析物易溶于氘代试剂，内标物化学结构稳定，具有溯源性，且不与待测化合物反应。OPO极性低，易溶于氘代氯仿，在氘代DMSO和氘代甲醇中的溶解性差，因此选择氘代氯仿作为溶剂。考察了尼泊金乙酯和苯甲酸两种具有溯源性的内标物，发现苯甲酸不溶于氘代氯仿，尼泊金乙酯可溶于氘代氯仿，苯环氢原子的化学位移6.88（见图6中A）满足定量要求。定量峰必须满足积分范围内无干扰、信号的化学位移尽可能接近内标物的信号峰、受到微量杂质的影响几率最小且基线平稳等条件，以减少误差。由于重叠峰、耦合裂分数较多、相邻质子峰分离条件不佳等因素将对NMR定值测量产生较大的误差，而OPO中油酸和棕榈酸上的两个质子峰（见图7中B）的化学位移值为2.33，且耦合裂分简单，质子峰之间不存在干扰，满足以上定量要求。通过比较尼泊金乙酯及OPO的核磁共振氢谱，确定添加内标尼泊金乙酯的OPO样品中OPO与尼泊金乙酯的化学位移分别为2.33和6.88，峰形尖锐且互不干扰，与相邻化学位移峰也无干扰。

**图6:**
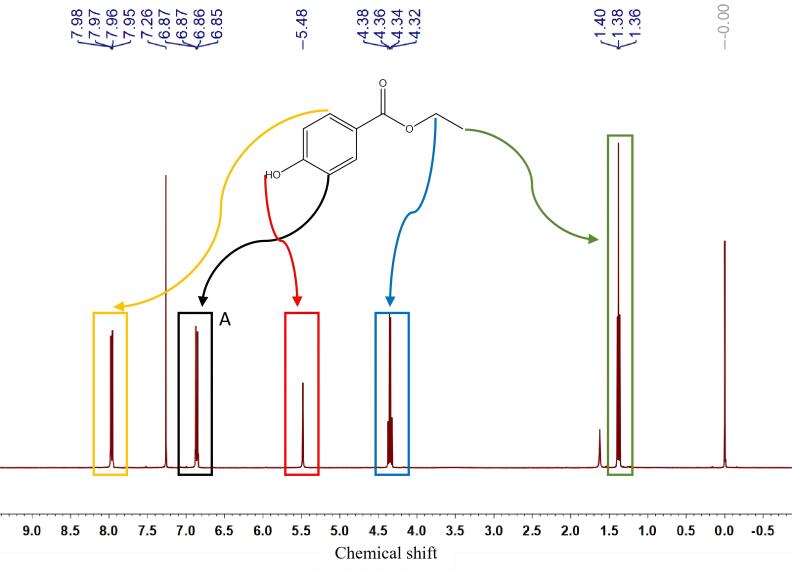
尼泊金乙酯在氘代氯仿中的核磁共振谱图

**图7:**
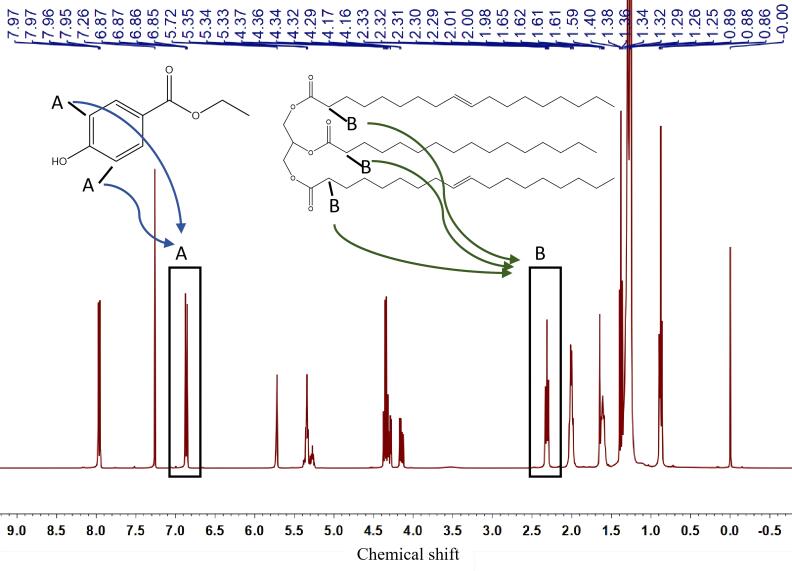
尼泊金乙酯和OPO混合物在氘代氯仿中的核磁共振谱图

#### 2.3.2 qNMR法定值结果

随机抽取6个OPO标准物质候选物，参照1.2.3节条件进行纯度测定。按国家计量技术规范JJF1855-2020 6.2节的公式计算OPO的纯度，结果见表2。由表2可知，qNMR法测定的OPO纯度值为98.82%。

**表2:** OPO纯度标准物质质量分数NMR定值结果

Sample	$\frac{I_{x}}{I_{std}}$	$\frac{n_{std}}{n_{x}}$	$\frac{M_{x}}{M_{std}}$		$\frac{m_{std}}{m_{x}}$	$P_{NMR}$
1	1.4587	0.3333	5.175		0.3956	99.02
2	1.4367	0.3333	5.175		0.4004	98.71
3	1.4266	0.3333	5.175		0.4027	98.58
4	1.4327	0.3333	5.175		0.4021	98.86
5	1.4079	0.3333	5.175		0.4083	98.64
6	1.4313	0.3333	5.175		0.4035	99.10
Average				98.82		
SD				0.21		

*I*_x_： integrated area of the specified peak of the sample； *I*_std_： integral area of the designated peak of the internal standard； *n*_std_： the number of nuclei of the designated peak for the internal standard； *n*_x_： the number of nuclei for the designated peak of the sample； *M*_x_： molecular weight of the sample； *M*_std_： molecular weight of the internal standard； *m*_std_： mass of the added internal standard； *m*_x_： weighed mass of the sample； *P*_NMR_： purity of the sample measured by qNMR.

### 2.4 OPO纯度标准物质定值结果

采用*F*检验考察质量平衡法与qNMR定值结果的离散程度，采用*t*检验验证两种方法测定平均值的一致性。检验结果表明两种方法的精密度接近、测定结果无显著性差异。因此采用两种不同原理方法定值结果的平均值作为OPO的纯度标准值，即98.67%。

### 2.5 不确定度评定

OPO纯度标准物质的不确定度由3部分组成：标准物质不均匀性引入的不确定度、标准物质不稳定性引入的不确定度和标准物质定值过程引入的不确定度。根据国家计量技术规范JJF 1343-2022《标准物质的定值及均匀性、稳定性评估》^［21］^，对OPO纯度标准物质进行均匀性检验、短期稳定性和长期稳定性考察，并计算不均匀性引入的不确定度*u*_bb_（瓶间均匀性不确定度）为0.027%、不稳定性引入的不确定度*u*_ls_（长期稳定性不确定度）和*u*_ss_（短期稳定性不确定度）分别为0.020%和0.030%。质量平衡定值方法主要评估杂质定量过程引入的不确定度，包括测量重复性等A类不确定度、称量和标准曲线计算等B类不确定度，由于杂质1、3的含量低于HPLC-CAD定值方法的定量限，因此将方法定量限（0.001 mg/mL）的20%作为3个杂质定量结果的不确定度来源之一，合成至标准曲线法定值引入的不确定度中，合成后得到该方法定值的不确定度*u*_MB_为0.351%；NMR定值主要评估测量重复性等A类不确定度以及称量、相对分子质量等B类不确定度，合成后的不确定度*u*_NMR_为0.230%。由于质量平衡法和NMR定量法的定值结果有一定差异，因此以平均值差的一半作为方法之间的差异引入的不确定度*u*_MB-qNMR_，为0.150%。

综上，合成OPO两种定值方法、定值结果差异、不均匀性和不稳定性等引入的不确定度，该纯度标准物质标准不确定度为0.33%，扩展不确定度为0.7%（*k*=2，*k*为包含因子，对应正态分布下约95%的置信概率）。

## 3 结论

本研究建立了OPO结构相关杂质的HPLC-CAD分析技术，基于CAD对同类结构类似物可表现出相近响应的特点，采用主成分对4个结构相关杂质进行准确定量，解决了无紫外吸收相关杂质定量的技术瓶颈，为甘油三酯类高纯标准物质的定值提供了新思路。同时本研究基于qNMR法，建立了OPO主成分的直接定值技术。两种不同原理定值技术的定值结果经检验无显著差异，一致性好，采用二者的平均值为OPO纯度标准物质赋值，标准值结果为98.7%，扩展不确定度为0.7%（*k*=2）。
